# Dataset of response of a semi anechoic room Frankonia SAC 3 plus driven by the electric field

**DOI:** 10.1016/j.dib.2018.09.072

**Published:** 2018-09-29

**Authors:** Martin Pospisilik, Stanislav Kovar, Vojtech Kresalek

**Affiliations:** Laboratory of Electromagnetic Compatibility, Faculty of Applied Informatics, Tomas Bata University in Zlin, Czech Republic

## Abstract

This document provides real data obtained inside the semi anechoic room Frankonia SAC 3 plus that is certified for measurement of electromagnetic interferences and performing of electromagnetic susceptibility tests in the range from 30 MHz up to 18 GHz. The hereby presented data describe the response of the semi anechoic room to the electric field driven inside. The frequency range of the experiment was from 10 MHz to 80 MHz where the standing waves are most likely to occur.

**Specifications table**

**Table:** 

Subject area	*Physics, Electronics*
More specific subject area	*Theory of electromagnetic field, Electromagnetic compatibility*
Type of data	*Figuers, MS Excel*
How data was acquired	*Isotropic field probe driving EMI test receiver*
Data format	*Raw and partially processed*
Experimental factors	*The measurement was obtained inside of the semianechoic room compliant with the standard CISPR 16. The same room and equipment is used extensively for electromagnetic compatibility tests and measurements*
Experimental features	*Electric field was excited inside the semianechoic room. Its frequency was changed from 10 MHz to 80 MHz, but the transmitting power was kept at a constant level. The intensity of the field was measured at 15 points inside the room for each of the transmitted frequencies.*
Data source location	*Zlin, Czech Republic (49.2304514 N, 17.6565100E)*
Data accessibility	*Data is with this article*
Related research article	*Pospíšilík, Martin; Soldán, Josef; Adámek, Milan. Influence of the properties of a real semi anechoic chamber on an internal electromagnetic field distribution. WSEAS Transactions on Systems, 2015, vol. 14., no.1, ISSN 1109–2777, 2224–2678**[11]*
	*Pospisilik, M., Silva, R., Adamek, M. Maple Algorithm for Damping Quality of Anechoic Chambers Evaluation, International journal of mathematics and computers in simulation. ISSN: 1998-0159, Volume 10, 2016**[12]*

**Value of the data**

• The data can help our colleagues get an idea of how the semi anechoic room Frankonia SAC 3 plus behaves in reality.
• The semi anechoic room Frankonia SAC 3 plus is widely used in many laboratories around the world. As its dimensions are given exactly, the expected dominant resonance frequency is as high as 27.77 MHz, according to the size of the biggest dimension. The data can serve as proof of the correctness of the calculation. At the frequencies around 27 MHz the effect of the standing wave between the most distant walls of the room is clearly visible.
• The data can be taken in account when the users of the Frankonia SAC 3 plus perform scientific experiments inside and need to establish a correction of the measured data.

## 1 Data

The MS Excel file contains raw data enlisted at the sheet “DATA”. When there is an electric field generated inside the semi anechoic room, its intensity can be measured. Although the transmitting power of the antenna is constant, the level of the electric field intensity measured at different points inside the room can be different. Moreover, the level is dependent on the frequency at which the field is driven. The sheet “DATA” contains a set of intensities measured at 15 different points inside the semi anechoic room that are marked by letters A to O. The values are provided in dBμV/m as this is the most used unit at the field of electromagnetic compatibility. At each point, the response of the room was measured for more than 600 different frequencies in the range from 10 to 80 MHz.

The file also contains the sheet “Legend”. At this sheet the displacement of the measuring point at the floor plan of the semi anechoic room is provided, so the reader can get orientation in the space of the room.

The last sheet is called “GRAPHS SPAN 2 MHz”. It contains a table that consists of averages of the raw data included at the sheet “DATA” made for frequency bands as wide as 2 MHz. The first line contains the average of the measured data for the centre of the band 20 MHz, which means that the average is calculated on the basis of the values obtained at the frequencies from 19 up to 21 MHz. The second line of the table refers to the band 22 MHz etc. Subsequently, 29 graphs are created on the basis of these data, showing how the displacement of the electromagnetic field varies according to the frequency. Each of the graphs is of a 3D type. The axes x any y refer to the position on the floor plan of the semi anechoic room while the z axis refers to the average of the field intensities in the framework of the relevant frequency band.

## 2 Experimental design, materials, and methods

The most usual spaces for performing of electromagnetic compatibility measurements are probably the anechoic and semi anechoic rooms the configuration of whose is in accordance with the standard CISPR 16 as amended. The performance of these rooms is determined by various physical phenomena. More information on these issues can be found in [10], [11], [12].

The aim of the experiment was to take an observation on how the residual standing waves inside the semi anechoic room Frankonia SAC-3 plus affect its performance.

### 2.1 Description of the semi anechoic room Frankonia SAC 3 plus

The semi anechoic room Frankonia SAC 3 plus is probably a typical semi anechoic room to be used for measurements with the distance between the measuring antenna and the transmitting object as long as 3 m. By covering or uncovering of its floor with pyramidal absorbers, the performance of the room can be changed from semi anechoic to fully anechoic and vice versa, according to the requirements of the appropriate experiment. For the purpose of gaining data published in this paper, the floor absorbers were removed and therefore the room acted as semi anechoic one. For example, this configuration is typical for measurements of electromagnetic interferences in the range from 30 MHz up to 1 GHz.

**Fig. 1:**
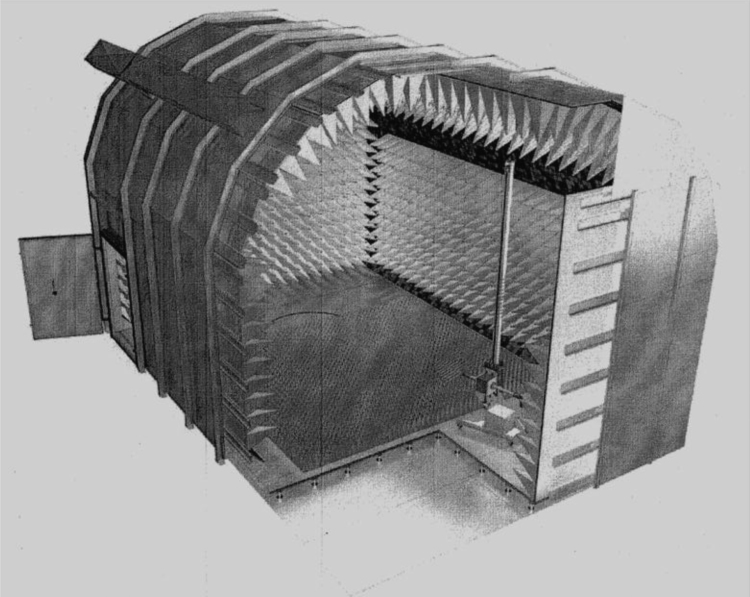
Semi anechoic chamber Frankonia SAC 3 – plus [4].

The shape of the room is depicted in the Fig. 1. Due to application of pyramidal absorbers mounted on the inner walls of the room, the effective inner floor dimensions are 8120 × 5150 mm. Maximum height of the cylindrically shaped ceiling is 9500 mm. More information on shielded rooms construction and usage can be found in the relevant literature [1], [2], [3], [6], [7], [8], [9].

### 2.2 Instrumentation

In order to perform the measurement, the following equipment was necessary:

– semi anechoic room Frankonia SAC 3 plus (described above),
– omnidirectional transmitting antenna (monopole) with a tripod,
– anisotropic spherical electric field probe HZ-11 [5] with a tripod,
– signal generator Rohde & Schwarz SMB 100 A,
– EMI test receiver Rohde & Schwarz ESU 8,
– controlling computer,
– set of coaxial cables.

### 2.3 Experiment setup

The experiment was processed inside the semi anechoic room. The signal generator and the EMI test receiver were, together with the controlling computer, placed outside the room.

**Fig. 2:**
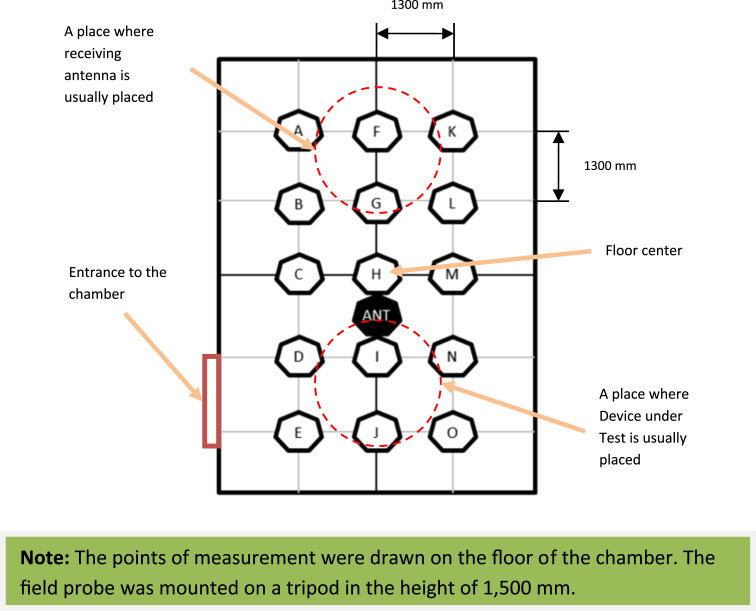
Displacement of the measurement points in the room.

The transmitting antenna was placed inside the room at the position depicted in the Fig. 2 where it is marked as ANT. It was connected to the signal generator by means of two coaxial cables. One of the cables was placed inside the room while the second one was placed outside. In order to obtain high shielding effectiveness of the room, the interconnection was performed by means of a penetration panel. The height of the transmitting antenna was adjusted in that way so its central point was 1500 mm above the room׳s floor.

The receiving anisotropic field probe was consequently placed at different points inside of the room. These points are also depicted in the Fig. 2, marked by the letters A to O. These letters correspond to the data file enclosed to this paper. Due to the internal dimensions of the room, the point H was marked in the centre of the floor and other points were determined symmetrically around it with a raster of 1300 mm. The field probe was mounted on a tripod and it was always placed in the height of 1500 mm. As well as the antenna, the probe was also connected to the receiver by means of two coaxial cables, interconnected by means of a penetration panel mounted on the wall of the room [4].

At each of the points, the frequency response of the room, the electrical field in which was driven by the antenna ANT, was measured with the same setup of the generator and the receiver. The data were stored in the data file which is enclosed to this paper.

**Fig. 3:**
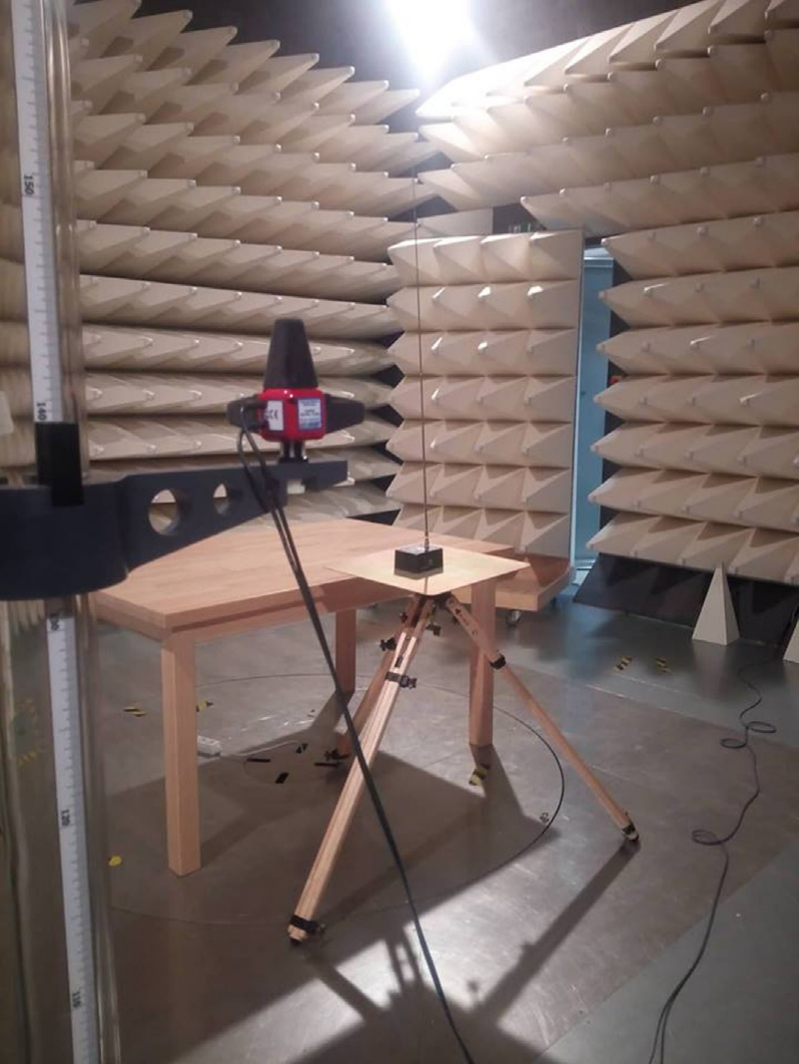
Experimental setup (example).

An example of the workplace setup (transmitting antenna and field probe inside the semi anechoic room) is depicted in the Fig. 3.

### 2.4 Determination of frequencies at which the measurement was processed

The frequency range of the semi anechoic room, guaranteed by its manufacturer, is 30 MHz to 18 GHz [4]. According to the dimensions of the room, its dominant resonance frequency is theoretically 27.77 MHz which was also confirmed by measurements [12]. The frequency range from 10 MHz to 80 MHz covers the most interesting frequencies around the dominant resonance of the room.

### 2.5 Generator and receiver setup

The generator and the receiver were not synchronized. The receiver was run as a spectrum analyser in fast frequency scanning mode and the max-hold mode, while the generator was slowly sweeping over the frequency range from 10 MHz up to 80 MHz. The frequency step of the sweep was 40 kHz. Together with the dwell time of 300 ms, the resulting measurement time for each of the points was 525 s.

The spectrum analyser was set in the following way: The resolution of 625 points led to a virtual frequency step of 112.179 kHz which is a final frequency step for data enlisted in the enclosed data set. The bandwidth was set to 120 kHz and the scanning period was 100 ms. With this setting, each of the frequencies produced by the generator was caught at least two times. Because the receiver run in the max-hold mode, the highest level was recorded for each of the frequencies.
